# Clearance of persistent HPV infection and cervical lesion by therapeutic DNA vaccine in CIN3 patients

**DOI:** 10.1038/ncomms6317

**Published:** 2014-10-30

**Authors:** Tae Jin Kim, Hyun-Tak Jin, Soo-Young Hur, Hyun Gul Yang, Yong Bok Seo, Sung Ran Hong, Chang-Woo Lee, Suhyeon Kim, Jung-Won Woo, Ki Seok Park, Youn-Young Hwang, Jaehan Park, In-Ho Lee, Kyung-Taek Lim, Ki-Heon Lee, Mi Seon Jeong, Charles D. Surh, You Suk Suh, Jong Sup Park, Young Chul Sung

**Affiliations:** 1 Department of Obstetrics and Gynecology, Cheil General Hospital and Women’s Healthcare Center, College of Medicine, Kwandong University, Seoul 100-380, Korea; 2 Research Institute, Genexine Inc., Korea Bio Park, Seongnam, Gyeonggi-do 463-400, Korea; 3 Department of Obstetrics and Gynecology, Seoul St Mary’s Hospital, College of Medicine, The Catholic University of Korea, Seoul 137-701, Korea; 4 Division of Integrative Bioscience and Biotechnology, Pohang University of Science and Technology, Pohang 790-784, Korea; 5 Department of Pathology, Cheil General Hospital and Women’s Healthcare Center, College of Medicine, Kwandong University, Seoul 100-380, Korea; 6 Department of Molecular Cell Biology, Samsung Biomedical Research Institute, Sungkyunkwan University School of Medicine, Suwon, Gyeonggi-do 440-746, Korea; 7 Laboratory of R&D for Genomics, Cheil General Hospital and Women’s Healthcare Center, College of Medicine, Kwandong University, Seoul 100-380, Korea; 8 Academy of Immunology and Microbiology, Institute of Basic Science, Pohang 790-784, Korea

## Abstract

Here, we demonstrate that electroporation-enhanced immunization with a rationally designed HPV DNA vaccine (GX-188E), preferentially targeting HPV antigens to dendritic cells, elicits a significant E6/E7-specific IFN-γ-producing T-cell response in all nine cervical intraepithelial neoplasia 3 (CIN3) patients. Importantly, eight out of nine patients exhibit an enhanced polyfunctional HPV-specific CD8 T-cell response as shown by an increase in cytolytic activity, proliferative capacity and secretion of effector molecules. Notably, seven out of nine patients display complete regression of their lesions and viral clearance within 36 weeks of follow up. GX-188E administration does not elicit serious vaccine-associated adverse events at all administered doses. These findings indicate that the magnitude of systemic polyfunctional CD8 T-cell response is the main contributing factor for histological, cytological and virological responses, providing valuable insights into the design of therapeutic vaccines for effectively treating persistent infections and cancers in humans.

## Abstract (web summary)

While several human papilloma virus (HPV) vaccines exist, a highly effective vaccine that mediates regression of HPV-induced tumours is lacking. Here the authors show that a therapeutic DNA vaccine-induced HPV-specific polyfunctional CD8 T cell in 7 out of 9 patients who all exhibited complete regression of lesions and viral clearance.

## Graphical abstract

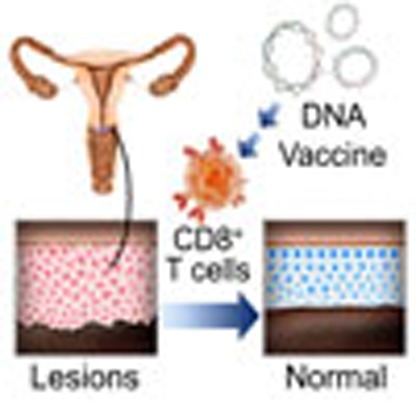

Cervical cancer is one of the leading causes of cancer death in women worldwide^1^,^2^, and about 75% of its cases are caused by persistent infection with the most common high-risk human papilloma virus (HPV) types, namely HPV16 and HPV18 (refs 3, 4). HPV persistence is usually associated with the lack of demonstrable HPV-specific T-cell immunity, and the virus-specific T cells found in pre-malignant and malignant patients are reported to be generally dysfunctional and sometimes even suppressive^5^,^6^. These findings suggest that the functional impairment of virus-specific T cells is a contributing factor for the emergence of HPV-induced cervical cancer.

The pre-malignant cervical intraepithelial neoplasia 2 and 3 (CIN2/3), in particular those positive for HPV16, are considered as high-grade lesions that have approximately a 30% chance of developing into invasive cancer^7^. Current treatment for CIN2/3 is limited to surgical excision, which is associated with about 10% recurrence rate and pregnancy-related complications, such as preterm delivery, low birth weight and premature rupture of membrane^8^. Recently introduced prophylactic HPV vaccines (Gardasil and Cervarix) have been shown to be effective in preventing HPV infection^9^, but without any therapeutic efficacy against pre-existing HPV infection or pre-malignant lesions. Therefore, there is an urgent need for an effective therapeutic vaccine that can eradicate HPV-related neoplasia without surgical manipulation.

HPV E6 and E7 act as viral oncoproteins by binding and promoting degradation of tumour suppressor proteins, p53 and retinoblastoma (pRb), respectively^10^. These viral oncoproteins are an ideal set of targets for a therapeutic vaccine against CIN2/3 and cervical cancer because these proteins not only induce tumorigenesis but also are constitutively expressed in HPV-infected pre-malignant and malignant cells^10^. Since the regression of cervical lesions is associated with the presence of a cellular, but not humoral immune response^11^,^12^, a therapeutic vaccine capable of selectively inducing robust E6/E7-specific T-cell immunity is highly desirable. Several attempts at this feat are currently underway with a varying level of success. Subcutaneous immunization with a recombinant vaccinia virus-expressing E6/E7 with interleukin-2 (IL-2) induced the regression of cervical lesions and cleared HPV infection in 10 out of 21 CIN2/3 patients^13^. Intrauterus immunization with another recombinant vaccinia virus-expressing bovine papilloma virus E2 elicited the regression of high-grade lesions in 19 out of 34 patients^14^. Unfortunately, none of these clinical studies presented whether the vaccine could induce a relevant T-cell immunity and elucidated the mechanistic explanation behind the observed therapeutic effect.

Induction of an HPV-specific T-cell response was observed with other vaccines, but without any meaningful clinical efficacy for the treatment of high-grade cervical lesions. One study found that subcutaneous immunization with an HPV16 E6/E7 synthetic long-peptide vaccine induced detectable level of HPV-specific interferon-γ (IFN-γ)-producing T-cell response in all five patients with high-grade cervical dysplasia, but without any reduction in HPV DNA and histological improvement of the cervical lesions^15^. This study resembles the past finding with intramuscular injections of an E6/E7 fusion protein mixed together with ISCOMATRIX adjuvant, which induced IFN-γ enzyme-linked immunospot (ELISPOT) responses in five out of 15 patients, but without any regression of the cervical lesions^16^. In clinical trials with DNA vaccines, intramuscular administration of an E7 DNA vaccine co-expressing HSP70 as a genetic adjuvant revealed measurable T-cell response in eight out of 15 patients with CIN2/3, although a histological regression was shown only in three out of nine patients^17^. In a recent study, E6/E7 DNA vaccine delivered via electroporation (EP) induced a significant HPV-specific IFN-γ-producing T-cell response in 14 out of 18 subjects, but the clinical response could not be examined because the enroled subjects had their cervical lesions surgically removed before vaccination^18^. In case of HPV-associated lower genital epidermal lesions, vaccination with a synthetic long-peptide vaccine induced T-cell response in all subjects and exhibited 47% of complete response rate in patients with vulvar intraepithelial neoplasia 3 (ref. 19). In essence, a highly effective therapeutic HPV vaccine that could induce more potent anti-HPV T-cell response and complete regression of high-grade lesions is yet to be clearly demonstrated in HPV-associated cervical neoplasia.

Here, we describe a newly developed HPV E6/E7 DNA therapeutic vaccine, designated GX-188, designed to facilitate the processing and presentation of HPV E6/E7 antigens by dendritic cells (DCs) through the co-expression of Fms-like tyrosine kinase-3 ligand (Flt3L). Vaccination with GX-188 by EP (GX-188E) elicits a significant E6/E7-specific T helper (Th) 1-polarized cellular immune response in all nine patients with CIN3. Importantly, GX-188E vaccination significantly induces polyfunctional HPV16-specific CD8 T-cell response in seven out of nine patients, which correlates with HPV clearance and complete resolution of high-grade cervical lesions on cytology and histology. Therefore, we offer an effective therapeutic vaccine strategy for eradicating persistent HPV infections and HPV-induced tumours, together with an important insight into the immune correlates to control persistent viral infection.

## Results

### GX-188E vaccination is safe and well tolerated

**Figure 1:**
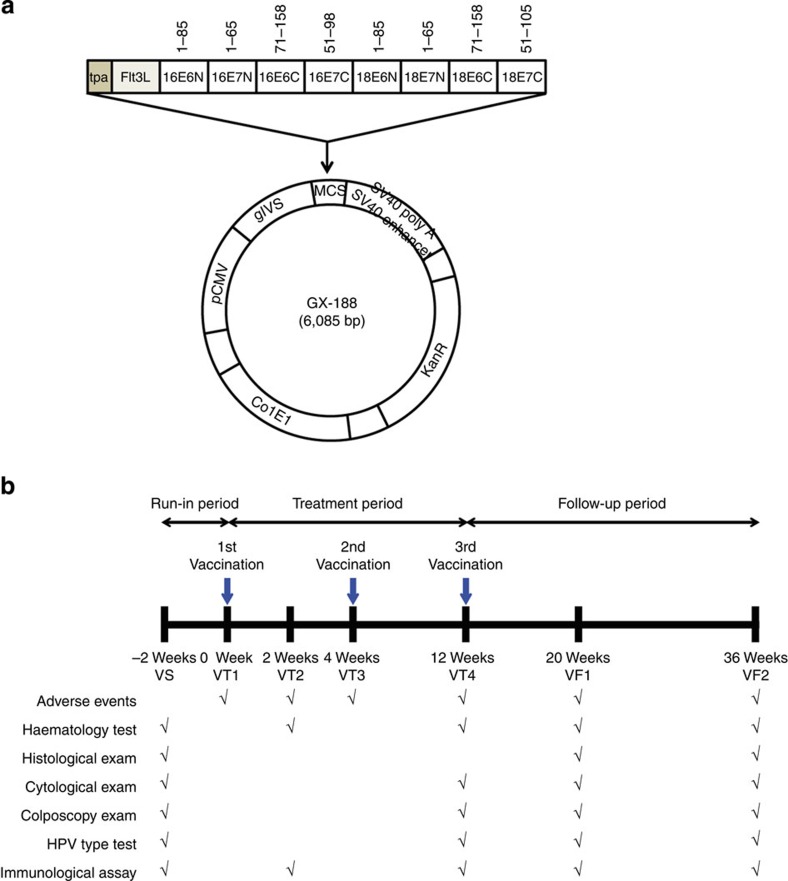
Diagram of GX-188 DNA vaccine and a schematic outline of the clinical trial. (**a**) GX-188 vaccine was constructed by inserting shuffled overlapping N- and C-terminal domains of E6 and E7 genes of HPV16 and HPV18 types into the pGX27 vector. The E6 and E7 domains are preceded by the secretory signal sequence of tpa and the extracellular domain of Flt3L. The inserted viral domains are abbreviated according to the HPV strain, the gene, and the domain; for example, 16E6N represents N-terminal domain of HPV16 E6. ColE1, ColE1-type bacterial origin of replication; gIVS, rabbit β-globin intervening sequence; KanR, kanamycin resistance gene; MCS, multi-cloning site; pCMV, cytomegalovirus early enhancer/promoter; SV40 poly A, Simian virus 40 late polyadenylation sequence; SV40 enhancer, Simian virus 40 enhancer. The numbers above each gene segment indicate the corresponding amino acid sequence. (**b**) The clinical trial had three periods: Screening of the recruited patients, treatment by three injections of the vaccine, and follow-up monitoring of the patients. Patients made visits for screening (VS), treatment (VT) and follow-up monitoring (VF) to the clinic during these three periods at the indicated time points to be examined and/or to receive vaccination.

GX-188 DNA vaccine is engineered to express E6 and E7 proteins of HPV16 and HPV18 fused to extracellular domain of Flt3L and the signal sequence of tissue plasminogen activator (tpa); the purpose for inclusion of Flt3L and tpa is to promote antigen presentation and trafficking of the fused protein to the secretory pathway, respectively (Fig. 1a). The activity of tpa is evident, as the GX-188-induced E6/E7 fusion protein was detected only in the cytoplasmic compartment of transfected cells, whereas E7 protein expressed by the same vector without tpa was found in both cytoplasmic and nuclear compartments (Supplementary Fig. 1a). The arrangement of HPV E6 and E7 genes in GX-188 was intentionally shuffled, by alternatively placing the amino (N-) and carboxyl-terminal (C-terminal) domains of the two genes, but with an overlapping region of 16 amino acids to minimize the loss of potential T cell-recognizing epitopes at the junction sites (Fig. 1a). The gene shuffling was done to prevent the homodimerization of E6 and E7 regions of the fusion protein, which is crucial for their binding and degradation of p53 and pRb tumor suppressor proteins^20^,^21^. As expected, the E6/E7 fusion protein generated by GX-188 DNA vaccine was unable to degrade p53 and pRb proteins, whereas wild-type E6 and E7 proteins induced their degradation (Supplementary Fig. 1b,c).

According to the inclusion and exclusion criteria for this study, nine out of 11 screened patients with CIN3 were enroled (Table 1). The screened patients were examined by multiple methods, including colposcopy, cytology, histology and HPV type test, at the visit for screening (VS) time point, 2 weeks before the start of the trial. All participating subjects received three injections of GX-188E, with the last two injections given at 4 and 12 weeks after the first injection (Fig. 1b).

A total of 49 adverse events (AEs) were recorded during all visits. Twenty-three AEs, including eczema, ecchymosis, vaginal itching, sleepiness, anorexia and dizziness were determined to be unrelated to the vaccination. Nineteen AEs including chills, injection site pain, swelling and hypoaesthesia, were recorded to be associated with GX-188E vaccination (Supplementary Table 1). Although the cause of the remaining seven AEs, including headache, rhinitis and fatigue, were unknown, they could be potentially associated with GX-188E vaccination. However, all these AEs were considered to be mild (grade 1) and all patients recovered completely within 3 days after GX-188E vaccination. Since neither severe AEs nor laboratory abnormalities were observed at any given dose (Supplementary Tables 1 and 2), the dose of GX-188E was elevated from 1 to 2 mg, and then to 4 mg (three patients at each dose) without the enrolment of additional three subjects at each dose level according to 3+3 dose-escalation design of this clinical trial protocol.

Since it was reported that the administration of Flt3L protein could increase the frequency of white blood cells (WBCs)^22^,^23^, we measured the number of WBCs and the level of Flt3L in the blood. A change in the number of WBCs was not observed (Supplementary Table 2), which is likely due to no significant upregulation of Flt3L level in the blood on GX-188E vaccination (Supplementary Table 3). Such a finding is in line with previous reports with incorporated genetic adjuvants in DNA vaccines, such as cytokines and HSP70 (refs 17, 24, 25, 26), implicating that the use of genetic adjuvants in a DNA vaccine is not usually associated with severe AEs.

To determine the immunological safety of our approach, we investigated whether the enhanced delivery of GX-188E generated anti-DNA antibodies, which are known to be associated with autoimmune disorders^26^. The level of antibodies against DNA in the blood of patients with CIN3 was below the detection limit (Supplementary Table 4), which is comparable to the previous results obtained from subjects immunized with DNA vaccine without EP^27^,^28^. Taken together, these results indicate that the incorporation of EP and genetic adjuvants is relatively tolerable in clinical trials of DNA vaccines and shows very similar safety profiles observed with the administration of a basic DNA vaccine without EP.

### GX-188E vaccination induces strong cellular immunity

**Figure 2:**
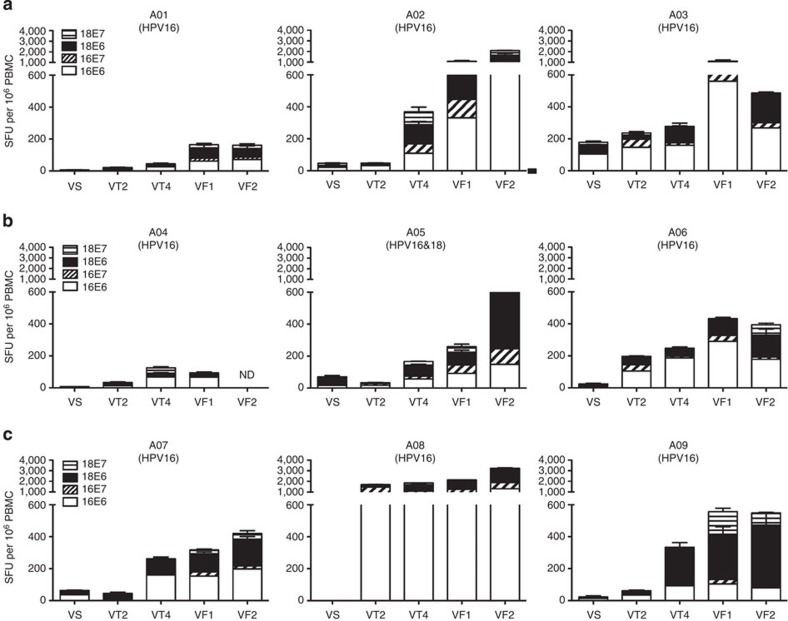
GX-188E vaccination induced significant HPV16 and HPV18 E6/E7-specific IFN-γ responses. Patients’ PBMCs were harvested and cryopreserved before (VS), during (VT2, VT4) and after (VF1, VF2) vaccination with GX-188E in all patients. The number of HPV16/18 E6- and E7-specific IFN-γ secreting cells in PBMCs was determined individually by IFN-γ ELISPOT assays after stimulation with HPV16 or HPV18 E6 and E7 peptide pools for 48 h at indicated time points in the 1 (**a**), 2 (**b**) and 4 mg (**c**) cohorts. Shown are the average spot-forming units (SFU) per 10^6^ PBMCs in triplicate wells against each antigen after subtracting the background number of spots (5.7±2.2). Error bars represent s.d. The percentage of E6-specific response in total number of spots was 76.6% (A01), 69.3% (A02), 88.9% (A03), 89.2% (A04), 69.1% (A05), 89.4% (A06), 84.2% (A07), 75.1% (A08) and 70.1% (A09) at VF1. The HPV types found in each patient are indicated in the parentheses. ND, not determined.

To study the cellular immune response induced by GX-188E, an IFN-γ ELISPOT assay as described in the Methods was performed before, at VS time point (−2 weeks), during, at VT2 (2 weeks) and VT4 time points (8 weeks), and after, at VF1 (20 weeks) and VF2 time points (36 weeks), GX-188E vaccination. Relatively high pre-existing IFN-γ ELISPOT response was detected in one patient (A03), whereas the other eight patients displayed weak pre-existing HPV-specific cellular immunity before vaccination. On the basis of the criteria described in the Methods, all subjects exhibited a marked increase in the vaccine-induced E6- and E7-specific IFN-γ ELISPOT response compared with the background level before vaccination (Fig. 2). Interestingly, two out of nine patients (A06 and A08) developed a considerably enhanced IFN-γ response even after a single immunization (VT2), and additional five patients (A02, A04, A05, A07, and A09) exhibited such an elevated response after two vaccinations (VT4). The remaining two patients (A01 and A03) in the 1-mg dose group displayed an increased IFN-γ response after three shots of the GX-188E vaccine (VF1). Taken together, these results suggest that vaccine-induced cellular immune responses became progressively stronger in all patients during GX-188E vaccination. In particular, patient A08 exhibited the highest magnitude of IFN-γ ELISPOT response with reactivity up to 3,500 spot-forming unit per 10^6^ peripheral blood mononuclear cells (PBMCs). Interestingly, the response against the E6 antigen was more vigorous than against E7 as determined by the magnitude of response (69–89% against E6 versus 11–31% against E7 at VF1; Fig. 2).

**Figure 3:**
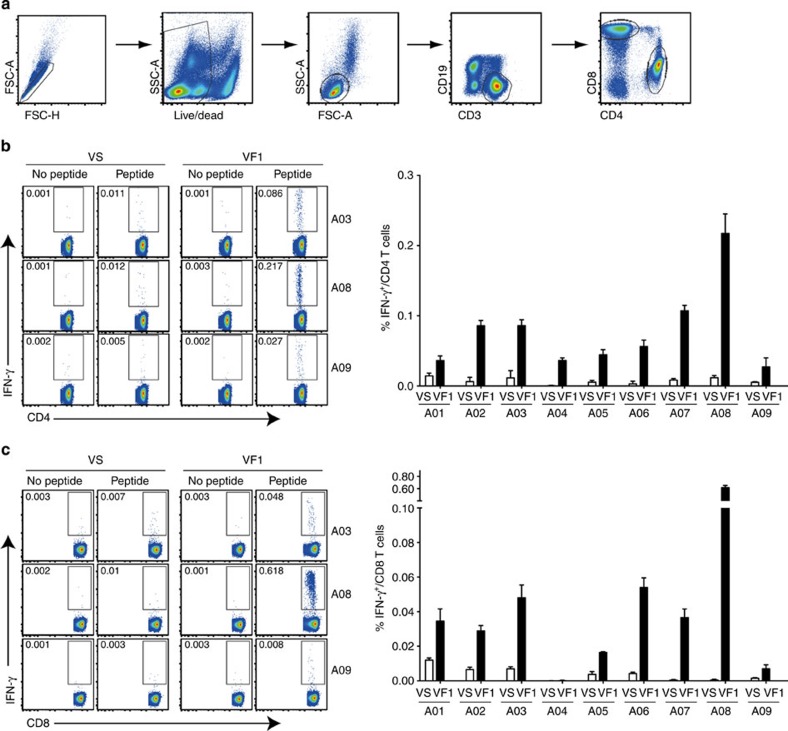
GX-188E vaccination elicited a significant increase in the frequency of HPV16-specific IFN-γ^+^ CD4 and/or CD8 T cells. Cryopreserved PBMCs of patients harvested before (VS) and after (VF1) GX-188E vaccination were stimulated with a combined mixture of HPV16 E6 and E7 peptide pools for 13 h. The frequency of HPV16-specific IFN-γ^+^ CD4 and CD8 T cells was determined by ICS followed by multicolour flow cytometry analysis. The gating strategy used to determine the IFN-γ-producing CD4 and CD8 T cells by flow cytometry (**a**) and the representative plots and the summary graphs showing the frequencies of IFN-γ^+^ CD4 (**b**) and IFN-γ^+^ CD8 (**c**) T cells before (VS) and after (VF1) vaccination. Data shown in the graphs represent the average of two independent experiments, with duplicate in each experiment, and error bars represent s.d. The background values were determined by the response of the medium only as a control and were ≤0.004±0.002% for CD4 and ≤0.003±0.002 for CD8 T cells (mean±s.d.).

The establishment of memory T cells, normally starting to form about 4 weeks after immunization, is usually one of the indispensable factors for protective efficacy of a vaccine^29^,^30^. A relatively high level of IFN-γ ELISPOT response was observed in eight out of nine patients at 24 weeks (VF2) following the last vaccination, which, when compared with the responses at 8 weeks (VF1) post vaccination, is decreased for one patient (A03), comparable for three patients (A01, A06, and A09) and increased for four patients (A02, A05, A07, and A08; Fig. 2). Overall, this finding indicates that GX-188E vaccination-induced E6/E7-specfiic memory T-cell response can be maintained for at least 24 weeks post last vaccination. To address whether the IFN-γ response to E6/E7 antigens measured by ELISPOT assay was generated mainly by T cells and to determine which subset of T cells played a predominant role, we performed intracellular cytokine staining (ICS) assays for IFN-γ at pre- and post-vaccination time points (VS and VF1). As shown in Fig. 3, the vaccination with GX-188E resulted in an increase in HPV16-specific IFN-γ^+^ CD4 T-cell responses in all nine patients, while IFN-γ^+^ CD8 T-cell response was enhanced in eight out of nine patients. Thus, with the exception of patient A04, GX-188E vaccine elicited activation of both HPV16-specific CD4 and CD8 T cells.

**Figure 4:**
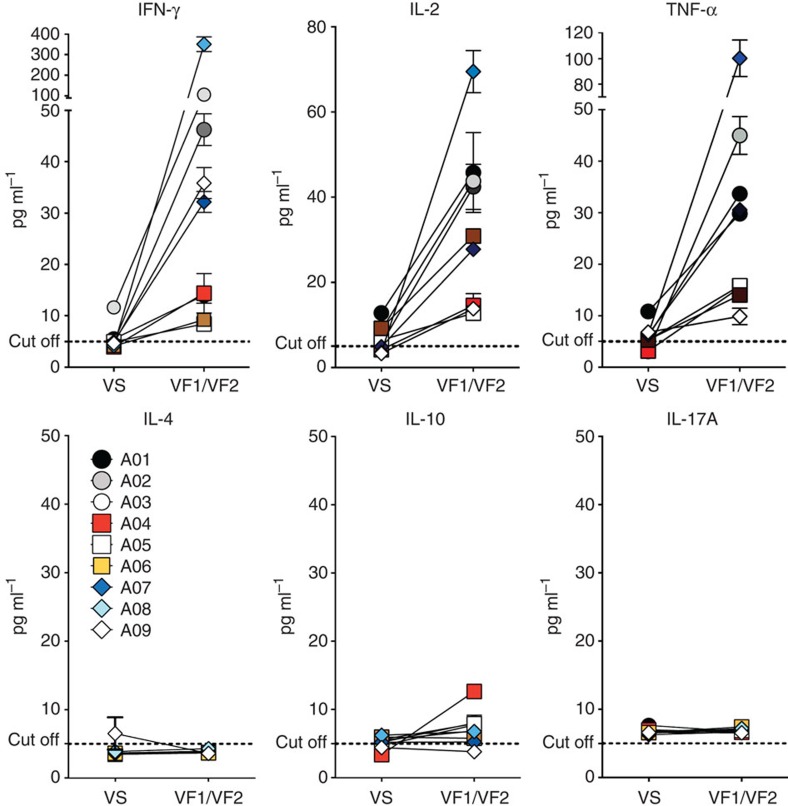
GX-188E immunization generated HPV16-specific Th1, but not Th2 or Th17 response. Cryopreserved PBMCs from patients before (VS) and after (VF1+VF2) vaccination were stimulated with a mixture of HPV16 E6 and E7 peptide pools for 48 h. Pooled PBMCs at VF1 and VF2 were used for all patients except for patient A04 in whom VF1 cells were used, as she received surgery before VF2. The indicated cytokines in the supernatants of cultures were quantified using Th1/Th2/Th17 cytometric bead array kit. Shown are mean±s.d. of triplicate. The horizontal dashed line indicates the cutoff background level determined by standard curve of each cytokine. Mean value of the medium alone background (mean±s.d., pg ml^−1^) was 4.19±0.41 for IFN-γ, 5.11±0.63 for IL-2, 5.58±0.88 for TNF-α, 3.3±0.24 for IL-4, 5.01±0.64 for IL-10 and 5.45±0.28 for IL-17A.

Since persistent HPV infection impairs Th1 cellular response to HPV, leading to cervical cancer progression^11^,^31^,^32^,^33^, we investigated whether our DNA vaccine could drive the differentiation of HPV-specific CD4 T cells into Th1 effector cells. As expected, the baseline production of common Th1 effector cytokines, such as IFN-γ, IL-2 and tumour necrosis factor-α (TNF-α), before vaccination was remarkably low on stimulation with E6/E7 peptides. However, the amounts of these cytokines markedly increased after vaccination in most of the patients (median 49.9-, 13- and 22.9-fold increases for IFN-γ, IL-2, and TNF-α, respectively; Fig. 4). Consistent with the IFN-γ ELISPOT and ICS data, A08 patient also showed the greatest increase of Th1 cytokine production. On the other hand, Th2 (IL-4 and IL-10) and Th17 (IL-17A) cytokines were not significantly increased by vaccination, although patient A04 had a slightly increased level in production of an immunosuppressive cytokine, IL-10 (Fig. 4). Taken together with the above IFN-γ ELISPOT and ICS analyses, these results suggest that GX-188E vaccination leads to the induction of a strong Th1-polarized HPV-specific cellular immune response.

### GX-188E vaccine-induced CD8 T cells are polyfunctional

**Figure 5:**
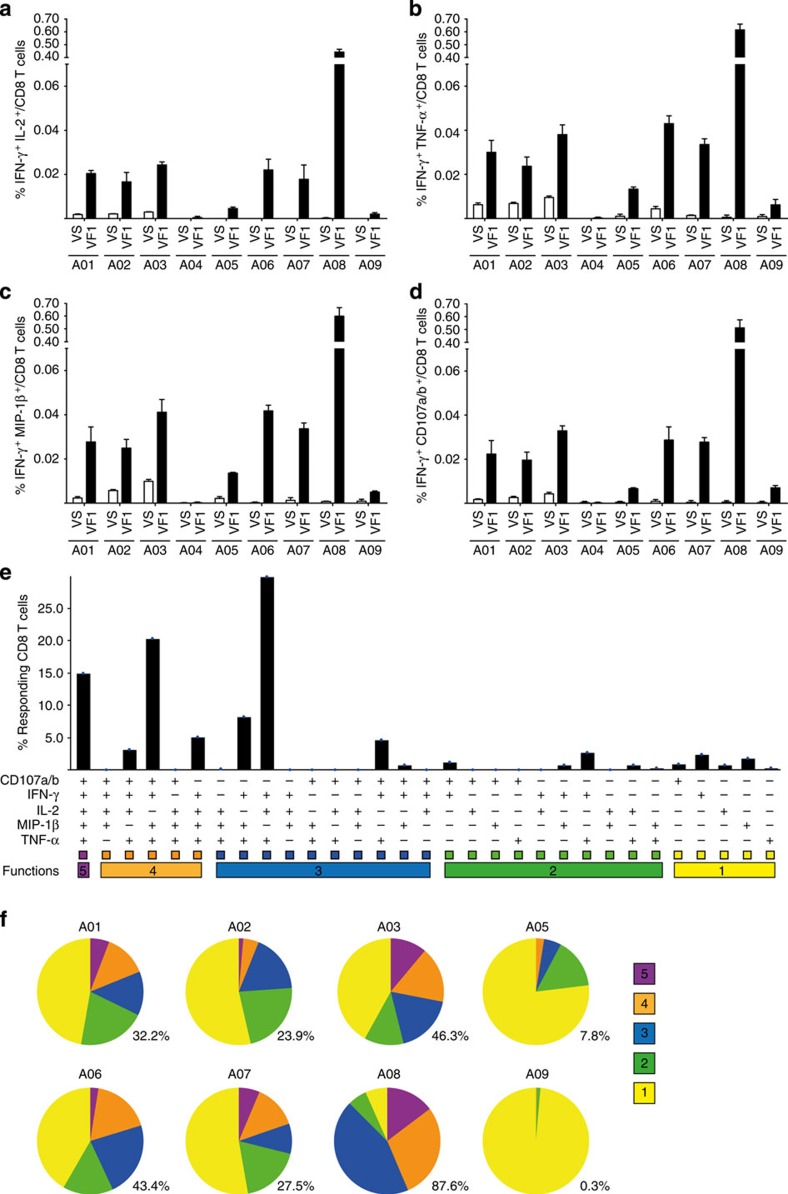
GX-188E vaccination induced the polyfunctionality of HPV16-specific CD8 T cells. Patients’ PBMCs were stimulated at before (VS) and after (VF1) vaccination as described in Fig. 3 and then analysed with multicolour flow cytometry to detect HPV16-specific expression of IL-2, IFN-γ, TNF-α, MIP-1β and the cytotoxic degranulating marker, CD107a/b. (**a**–**d**) The summary graphs show the frequencies of IFN-γ^+^ CD8 T cells co-expressing IL-2 (**a**), TNF-α (**b**), MIP-1β (**c**), CD107a/b (**d**) on gated CD8 T cells. (**e**) Representative graph of A08 patient’s polyfunctional responses to HPV16 E6/E7 peptides subsequent to Boolean gating after vaccination (VF1). The five functions, CD107a/b, IFN-γ, IL-2, MIP-1β and TNF-α are listed along *x* axis with each of their respective 31 possible combinations. The five horizontal bars of different colours below *x* axis depict the populations of five, four, three, two or one functional responses. (**f**) Each pie chart represents the relative frequency of HPV16 E6/E7-specific CD8 T cells with each combination of the five functional responses post vaccination (VF1). The numbers to the bottom right of each pie chart indicate the percentage of HPV16-specific CD8 T cells that produce three or more functional molecules. The polyfunctional profile of A04 patient was not available because of too low frequency of the responding CD8 T cells for analysis. Data shown in the graphs represent the average of two independent experiments, with duplicate in each experiment, and error bars represent s.d. The background value was determined by the response of the medium only and was 0.0008±0.001% for IFN-γ^+^ IL-2^+^, 0.0016±0.0014% for IFN-γ^+^ TNF-α^+^, 0.0015±0.0019% for IFN-γ^+^ MIP-1β^+^ and 0.0009±0.0012% for IFN-γ^+^ IL-2^+^ CD8 T cells (mean±s.d.).

To determine whether GX-188E vaccination induced multiple aspects of HPV-specific CD8 T-cell functionality, we assessed the ability of CD8 T cells, on stimulation with a pool of HPV16 E6 and E7 peptides, to simultaneously express the following five different effector functions: cytokines IFN-γ, IL-2 and TNF-α, a chemokine MIP-1β and the cytotoxic activity as determined by expression of CD107a/b, which is exclusively found during degranulation of cytotoxic T cells^34^. When T cells were examined for two effector functions (IFN-γ together with IL-2, TNF-α, MIP-1β or CD107a/b), we obtained results similar to that obtained by ICS for only IFN-γ. Thus, eight out of nine patients, with exception of A04, displayed an increase in proportions of HPV-specific CD8 T cells with two effector functions after vaccination (VF1) compared with before vaccination (VS) (Fig. 5a–d and Supplementary Fig. 2b–e). Examination of all five effector functions simultaneously as assessed using Boolean gating revealed even more striking results. Data on post-vaccination T cells from the most responsive patient (A08) are shown as an example in Fig. 5e, and together with the pie chart in Fig. 5f, shows that 87.6% of HPV16-specific CD8 T cells were at least triple-positive and 15% of them had all 5 functions. Similar analysis in the other six patients (A01, A02, A03, A05, A06 and A07) revealed that 7.8–46.3% of HPV-specific CD8 T cells had three or more functions (Fig. 5f). In contrast, almost all HPV16-specific CD8 T cells from patient A09 were not polyfunctional (Fig. 5f). It is worth noting that Boolean gating analysis could not be performed with prevaccination T cells from all patients and post-vaccination T cells from patient A04 because of extremely low frequency of responding T cells that displayed effector function. Overall, these results indicate that GX-188E vaccination efficiently induced generation of polyfunctional HPV-specific CD8 T cells in most patients (seven out of nine patients).

**Figure 6:**
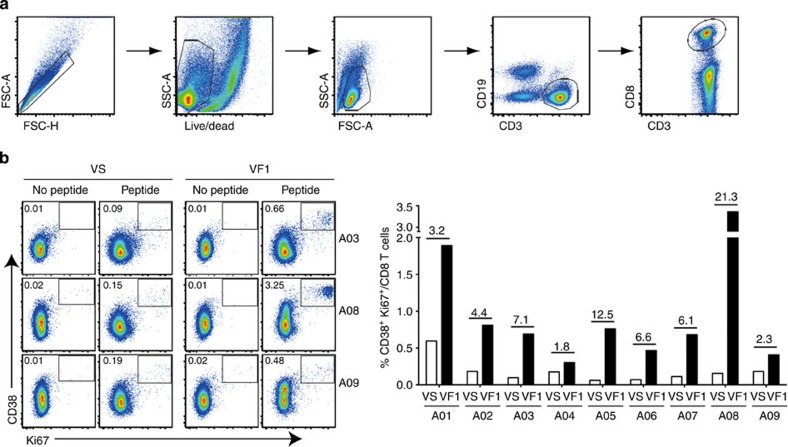
GX-188E vaccination induced proliferation of HPV16-specific CD8 T cells. Patients’ PBMCs were stimulated at before (VS) and after (VF1) vaccination and analysed by flow cytometry as described in the Methods to examine the expression of CD38 and Ki67 on virus-specific CD8 T cells. (**a**) Gating strategy to determine the expression Ki67 and CD38 on CD8 T cells by flow cytometry. (**b**) The representative plots and the summary of data show the frequency of proliferating CD38^+^ Ki67^+^ CD8 T cells. Data shown in the graph represent the average of two independent experiments. The cells shown in plots are gated on CD8 T cells. The numbers on the bar graph indicate fold increase post vaccination. The background value was determined by the response of the medium only control, which was 0.011±0.015% for CD38^+^ Ki67^+^ CD8 T cells (mean±s.d.).

It was reported that optimal expansion of responding T cells on antigen stimulation is essential for providing effective protective immunity by therapeutic vaccination^35^,^36^. Therefore, we examined the proliferative potential of HPV-specific CD8 T cells by stimulating patients’ PBMCs from pre- (VS) and post- (VF1) vaccination with a pool of HPV16 E6/E7 peptides for 5 days, followed by staining for the expressions of Ki67 and CD38, which serve as a marker of proliferation and activation, respectively^37^,^38^. PBMCs stimulated with medium alone served as a negative control to assess antigen specificity of *in vitro*-expanded CD38^+^Ki67^+^ CD8 T cells. Although one patient (A01) displayed a relatively high pre-existing proliferative level prevaccination (VS), the rest of the patients demonstrated low levels of Ki67^+^CD38^+^ CD8 T cells (Fig. 6). After vaccination, all patients exhibited meaningful improvement in proliferative activity of HPV-specific CD8 T cells. In accordance with the pattern of functional CD8 T cell response (Fig. 5), two patients (A04 and A09) displayed only a minor increase in proliferating CD8 T cell population, whereas the other seven patients displayed a much greater increase of Ki67^+^CD38^+^ CD8 T-cell population, within a range of 3.2- to 21.3-fold increase. Collectively, these results indicate that GX-188E vaccination in CIN3 patients substantially augmented both the expansion and polyfunctionality of HPV-specific CD8 T cells.

### GX-188E elicits a weak antibody response to E7 protein

When plasma samples were evaluated for total IgG antibody responses to E6 and E7 by an end point dilution enzyme-linked immunosorbent assay, all patients had barely detectable or undetectable IgG titer to both E6 and E7 proteins at baseline (VS; Supplementary Fig. 3), indicating no meaningful pre-existing E6- and E7-specific IgG antibody responses. Interestingly, the antibody titres to E6 were not induced in any dose cohort patients after vaccination. Three out of nine patients (A05, A07, and A09) generated weak anti-E7 antibody responses following vaccination with antibody titres ranging from 1:8 to 1:256 (Supplementary Fig. 3). It is worth noting that T-cell responses to E7 antigens were generally lower than those against E6 antigens (Fig. 2) and measurable antibody titres to E7 proteins were not associated with CD8 T cell responses to E7 antigens.

### GX-188E vaccination clears HPV infections and lesions

**Figure 7:**
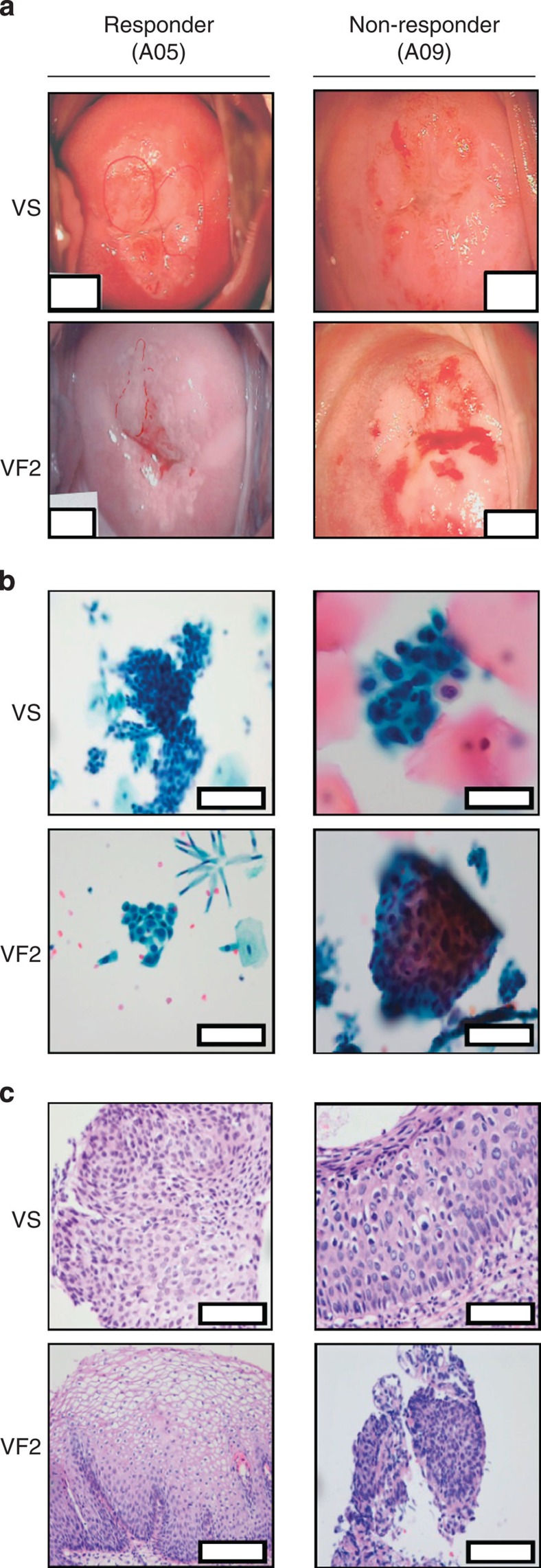
GX-188E vaccination led to the clearance of cervical lesions as determined by colposcopy, cytology and histology. Photographs of cervical colposcopy (**a**), endocervical cytology (**b**) and histology (**c**) from a representative responder (A05) and a non-responder (A09) patient before (VS) and after (VF2) GX-188E immunization are shown. (**a**) Patient A05 at VS exhibited dense acetowhite epithelium with coarse punctuation in transformation zone, but at VF2 showed reduced intermediate acetowhite epithelium without punctuation; patient A09 at VS and VF2 displayed dense acetowhite epithelium with rolled margin and coarse punctuation in transformation zone. Colposcopic pictures of the cervix at × 6 magnification were taken. The white bars are used to prevent the disclosure of patients’ information. (**b**) Patient A05 at VS exhibited high-grade squamous intraepithelial lesion (HSIL) with enlarged nuclear size and hyperchromasia, but at VF2 showed only normochromic epithelium with no intraepithelial lesion (NIL). Scale bars, 10 μm; patient A09 at VS and VF2 displayed HSIL variable nuclear size and hyperchromasia. Scale bars, 5 μm. (**c**) Patient A05 at VS was diagnosed as CIN3 with full thickness of the epithelium, and with mitoses visible in the upper layer, but at VF2 displayed normal squamous epithelium without atypical neoplastic cells. Scale bars, 40 μm; patient A09 at VS and VF2 was diagnosed as CIN3 with thick and abnormal epithelium and the presence of keratinized cells with nuclear atypical in the upper layer. Scale bars, 20 μm.

GX-188E-induced clinical responses were determined by evaluating the patients’ HPV infection status as well as the cytological and histological changes of their high-grade cervical lesions over the 36-week period of the clinical trial (Table 2 and Fig. 1b). At baseline (VS), all nine patients had CIN3 with either severe dysplasia (A01, A02, A05, A06, A07 and A08) or carcinoma *in situ* (A03, A04 and A09) according to the histological evaluation of colposcopic-directed biopsy specimens (Tables 1 and 2). At 8 weeks post last vaccination (VF1), six out of nine patients were free of lesions—two patients from each cohort (A01 and A03 from 1 mg cohort, A05 and A06 from 2 mg cohort, A07 and A08 from 4 mg cohort)—indicating dose independency of the response presumably due to saturation dose at 1 mg (Table 2). Three of these responder patients (A03, A06 and A08) were negative for the intraepithelial lesion based on cytological analysis after the second immunization at week 12 (VT4), while three other patients (A01, A05 and A07) displayed such responses after the third vaccination at week 20 (VF1) and the last responder patient (A02) cleared the lesion at the end of the 36-week trial (VF2). Notably, none of the six early responders displayed any recurrent cervical dysplasia during the remaining period of the trial. In cases of two non-responders, patient A04 was treated by cervical conization at week 24, while patient A09 was monitored without surgery until the end of study per patient’s request and remained stable at CIN3 without progressing to invasive carcinoma. Colposcopic, cytological and histological image analysis before vaccination (VS) and at the end of the trial (VF2) more clearly demonstrated the difference in clinical responses to GX-188E between responders and non-responders, as shown by the photographs from representative responder A05 and non-responder A09 patients (Fig. 7).

HPV16 was identified in the lesions of all nine subjects at the start of the trial, and one patient (A05) was found to be also co-infected with HPV18. At week 12 (VT4), four patients (A01, A03, A06 and A08) and patient A05 showed clearance of HPV16 and HPV18 viruses, respectively (Table 2), indicating viral clearance after the second immunization. At week 20 (VF1), HPV DNAs in cervical lesions were cleared in six out of nine patients (A01, A03, A05, A06, A07 and A08) and one more patient (A02) cleared the virus at week 36 (VF2). Since these seven patients also cleared their lesions with the identical kinetics, there was perfect correlation between the clinical and virological responses (Table 2). Beside HPV16 and HPV18, two patients (A06 and A07) were found to be co-infected with other high-risk common types of HPV at baseline (VS). In addition, one patient (A05) became infected with the common HPV type in the midst of the trial (VT4). In contrast to A07 patient, A05 and A06 patients cleared co-infected common types of HPV at VF2 and VT4, respectively, presumably due to a bystander effect caused by the elimination of HPV16-infected intraepithelial neoplastic cells.

It is notable that the three patients (A03, A06 and A08) who cleared their lesions and HPV infection at the early time point (VT4) promptly displayed a relatively high magnitude of HPV-specific polyfunctional CD8 T-cell response (Table 2 and Fig. 5). In addition, the other four patients (A01, A02, A05 and A07) with a meaningful polyfunctional CD8 T-cell response exhibited the complete resolutions of their lesions and HPV infections after the third vaccination either at week 20 (VF1) or at the end of the trial (VF2; Table 2 and Fig. 5). In contrast, two non-responder patients (A04 and A09) had almost no polyfunctional CD8 T-cell response. The correlation between the induction of polyfunctional T-cell response and clinical outcome is readily apparent when the individual data from the patients were grouped into non-responders (A04 and A09) and responders (A01, A02, A03, A05, A06, A07 and A08) to generate the polyfunctional profile with three or more functions (Supplementary Fig. 4). Hence, our results indicate that the clinical efficacy of GX-188E vaccine strongly correlates with the extent of systemic HPV-specific polyfunctional CD8 T-cell response. Overall, GX-188E vaccination led to the clinically and virologically meaningful complete response rate of 78% (seven out of nine patients) (Table 2).

## Discussion

In this study, we have demonstrated the efficacy of a novel HPV E6/E7 DNA therapeutic vaccine, designated GX-188, strategically designed to induce robust type 1 T cell-mediated immunity and to eradicate HPV infection-associated lesions. So far, several types of HPV therapeutic vaccines have been evaluated in patients diagnosed with CIN2/3 lesions using viral vectors^13^,^14^, peptides^15^, proteins^16^ or plasmid DNA^17^. In contrast to these previous studies that performed excision surgery at weeks 9–15 after vaccination, preventing extended observation of histological change, our current trial was designed to assess the clinical outcomes and immune responses at 20 and/or 36 weeks after the first vaccination to investigate the kinetics of immune response and the durability of vaccine efficacy. In this study, viral clearance (four out of nine patients) and cytological recovery (three out of nine patients) were already apparent at week 12 and most of the complete responders (six out of seven patients) cleared the cervical lesions within week 20 post vaccination with durable immunity, that is, without any recurrence of cervical dysplasia or re-emergence of HPV infection during the remaining 16 weeks period of the clinical trial. Collectively, compared with the previous reports, our results appear to be highly novel and clinically meaningful for the following reasons: first, the EP-delivered GX-188 elicited the relatively high and durable HPV-specific Th1-polarized cellular immune responses in eight out of nine CIN3 patients with severe dysplasia or carcinoma *in situ*, and seven out of nine patients (78%) had complete regression of their high-grade lesion and clearance of HPV DNA, which are the highest response rate compared with the previous reports in clinical trials of HPV vaccines. Second, cytological, histological and virological evaluations yielded the same results in the responders, indicating induction of a complete response by GX-188E. Third, the normalization of lesions by GX-188E vaccination was maintained without recurrence of dysplasia during the entire study period of 36 weeks, indicating a sustained therapeutic efficacy of the vaccine. Finally, clinical and virological responses at the cervix were correlated with the magnitude of the polyfunctional CD8 T-cell response in the blood, suggesting that systemic HPV-specific CD8 T-cell responses might serve as a predictive biomarker for determining clinical outcomes of therapeutic vaccine in the setting of high-grade cervical lesions.

It has been reported that regression of CIN2/3 can occur spontaneously in some patients at a rate ranging from 11 to 30% over ≥1 year to 38% between 9 and 20 weeks after colposcopic biopsy^39^,^40^,^41^. Even when considered in light of these reports, our finding of complete regression in six out of nine patients (67%) at 20 weeks and in seven out of nine patients (78%) at 36 weeks after GX-188E vaccination indicates that vaccine-induced immunity is considerably more effective than natural immunity. However, we cannot rule out the possibility that some of the responders, especially the ones who have responded early, would have spontaneously cleared their lesions. A corollary to this possibility is that the vaccine-induced response rate beyond the maximum rate of spontaneous regression is in the same ballpark as that of landmark vaccine trial in which a synthetic long-peptide vaccination exhibited 47% of complete response rate in patients with another HPV-associated lower genital dysplasia, vulvar intraepithelial neoplasia 3 (ref. 19).

The induction of strong HPV-specific Th1 and CD8 T-cell immunity by GX-188E vaccination leading to complete clearance of HPV DNA and high-grade cervical lesions observed in this study is likely due to the following reasons: codon optimization of HPV E6/E7 genes^42^, intracellular targeting of expressed E6/E7 antigens to secretion pathway by the fused tpa signal sequence^43^, enhanced DNA vaccine delivery by EP^44^ and the utilization of a high-expression vector^45^. In addition, since mutations or deletions of the E6/E7 sequence motifs crucial for binding to p53 and pRb could result in loss of their T cell-recognizing epitopes^17^,^18^, we instead genetically engineered HPV E6 and E7 genes by shuffling N- and C-terminal domains, with an overlapping region, to maintain T-cell epitopes, while abrogating the oncogenic potential of these proteins. Finally, the Flt3L gene was fused to engineered E6/E7 genes as a built-in genetic adjuvant, since Flt3L is known to induce Th1 immune responses through preferential expansion of CD8α^+^ lymphoid DCs^46^,^47^,^48^,^49^. It was reported that bone marrow-derived DCs pulsed with cell lysates containing Flt3L-E7 fusion protein presented E7 antigens through the major histocompatibility complex class I pathway more efficiently than those pulsed with wild-type E7 protein^50^, indicating a potential role of Flt3L in facilitating cross-priming. Moreover, Flt3L-fused DNA vaccine elicited the highest antigen-specific IFN-γ-secreting T-cell responses compared with other molecules for targeting antigens to DCs, such as CD40L and flagellin^43^,^51^. In contrast to T-cell responses, GX-188E vaccination induced the mild increase of IgG titres against only E7 protein in three out of nine patients (A05, A07 and A09; Supplementary Fig. 2). These weak antibody responses can be explained by the feature of GX-188 DNA vaccine in which Flt3L-mediated Th1-polarization may inhibit Th2-type responses and antibody production by cross-regulation of Th1/Th2 responses^52^. Since persistent HPV infection is known to promote Th2 response, thereby leading to poor T-cell immunity and continual progression of lesion^11^,^31^,^32^, our strategy to incorporate Flt3L to HPV DNA vaccine antigen may be an effective approach for inducing Th1-polarized cellular immunity.

The immunological and clinical outcomes after vaccination are generally variable and may be determined in part by host genetic factors. In the present study, GX-188E vaccine achieved complete response in seven out of nine patients (78%). Among seven responders, six patients carrying human leukocyte antigens (HLA)-A*02 exhibited high polyfunctional CD8 T-cell responses as well as complete regression of CIN3 (Table 1). Among the two non-responders, patient A04 with HLA-A*26 and -A*30 did not induce HPV-specific CD8 T-cell responses at all. It was reported that 5–15% of vaccine failed to seroconvert by the standard hepatitis B vaccination schedule and this was found to be associated with specific HLA alleles^53^. In this regard, it will be of interest to investigate whether specific HLA alleles can be a contributing factor for the impaired induction of HPV-specific CD8 T-cell response by GX-188E vaccination.

The other non-responder patient (A09) exhibited a comparable magnitude of antigen-specific CD8 T-cell response with two effector functions, but had lower polyfunctional profile of CD8 T cells with three or more effector functions than responder patients, indicating that the polyfunctionality of vaccine-induced CD8 T cells may contribute to clinical outcomes. It is worth noting that the patient A09, who was infected with only HPV16, displayed a higher T-cell response to HPV18 than to HPV16 in IFN-γ ELISPOT assay. Thus, this biased response towards HPV18 in the patient A09 may have led to the impaired HPV16-specific polyfunctional CD8 T-cell response. Alternatively, it is possible that local immunity at cervix could be involved in the clearance of lesions. Recently, combined vaccination with a heterologous DNA prime-recombinant vaccinia vector-based boost regimen induced a tissue-localized T-cell response in most patients with CIN2/3, which was suggested to be informative for determining vaccine efficacy^54^. In our current trial, complete clearance of lesions was already achieved at the first biopsy after vaccination (at week 20) in most patients, and thus we could not obtain the lesions to assess vaccine-induced local immunity. To address this issue, we are planning to take a biopsy at an earlier time point (week 6–8 after first vaccination) in upcoming phase 2 clinical trial. Finally, patient A09 had the largest lesion size with carcinoma *in situ*, probably providing more severe immunosuppressive microenvironment by which the effector function of vaccine-induced systemic CD8 T cells could be thwarted. It was reported that median cell density of stromal Foxp3^+^ regulatory T cells expressing IL-10 and indoleamine 2, 3-dioxygenase appear to increase significantly in the cervix with increasing pathology and cancer^55^. Thus, it would be important to develop strategies to neutralize these immunosuppressive factors and/or enhance the vaccine-induced T-cell responses to improve the clinical efficacy of DNA vaccines in patients with larger lesions and cancer.

Among three CIN3 patients with carcinoma *in situ*, one patient (A03) who had a relatively high pre-existing HPV-specific cellular immune response completely cleared both HPV infection and the high-grade lesions on GX-188E vaccination. Thus, it would be of interest to investigate whether the pre-existing cellular immunity could be an indicator of the clinical outcome of therapeutic vaccine in CIN3 patients with carcinoma *in situ*. Most importantly, all six CIN3 patients with severe dysplasia showed eradication of HPV DNA and regression of their lesion following GX-188E DNA vaccination. Even though the number of patients (n=9) is too small to draw a definite conclusion, our findings may provide an important insight into the key requirements for a therapeutic vaccine to be effective against various persistent infections and chronic diseases. Considering that the current primary treatment option for CIN3 is surgical excision, which is often accompanied with several side effects^8^, our non-invasive immunological approach with good safety and excellent clinical efficacy within 12 to 36 weeks could mark one of important milestones for treating CIN3 patients with severe dysplasia or carcinoma *in situ*.

## Methods

### Ethics statement

The clinical trial protocol was reviewed and approved by the Institutional Review Board at Cheil General Hospital and Women’s Healthcare Center (CGH-IRB-2012-35). This study was conducted in accordance with the ethical principles that had their origin in the current Declaration of Helsinki and was consistent with International Conference on Harmonization Good Clinical Practice (IHC GCP) and applicable regulatory requirements. All study participants gave written informed consent before undergoing screening for study eligibility and enrolment. This trial is listed at http://www.clinicaltrials.gov (NCT01634503)

### Study design and patients

This phase 1 clinical study was conducted as an open label, single center, dose-escalation study at Cheil General Hospital & Women’s Healthcare Center, Seoul, Korea. The primary end point was to evaluate safety and tolerability in patients with CIN3. The secondary end points included systemic induction of HPV E6- and E7- specific T-cell immune responses measured by IFN-γ ELISPOT, and changes of involved lesions and HPV infection status at the uterine cervix. Women aged between 20 and 50 years with histologically and virologically proven HPV16- or HPV18-associated CIN3 were enroled in the study. The CIN3 was confirmed by colposcopy-directed biopsy and HPV16 or HPV18 positivity was determined by PCR. Subjects with hepatitis B virus, hepatitis C virus or human immunodeficiency virus infections, abnormal electrocardiography including arrhythmia, history of severe adverse drug events or severe allergic diseases were excluded. Females who were pregnant or planning to be pregnant were not recruited in the study. Vaccination consisted of a series of three vaccine injections administered intramuscularly to alternating deltoid muscles at weeks 0, 4 and 12. A standard 3+3 dose-escalation scheme was followed and dose levels of 1, 2 and 4 mg were tested. At the highest dose, 4 mg of GX-188E was split into 2+2 mg and injected to the left and right deltoids muscles. For the intramuscular injector, an EP device (TriGrid Delivery System, Ichor medical systems, Inc.) was used to facilitate DNA uptake into cells.

### HLA typing

HLA typing was accomplished at Catholic Hematopoietic Stem Cell Bank, College of Medicine, The Catholic University of Korea, Seoul, Korea. Sequence-based typing (SBT) of HLA was performed by heterozygous amplification followed by sequencing of the complete exons 2, 3 of HLA-A and -B. For locus-specific amplification, primers were used in in-house method. After application by PCR, agarose gel electrophoresis of the PCR products was conducted to assess the quantity and quality. Cycle sequencing reactions using the ABI PRISM BigDye terminator kit (Applied Biosystems, CA, USA) and the automated ABI377 DNA Sequencer (Applied Biosystems, CA, USA) were performed. These data were analysed by using SBT analysis programme (Conexio Genomics, Assign SBT v3.5.1).

### DNA vaccine

The study vaccine, pGX-188, contains a plasmid DNA encoding E6 and E7 proteins of HPV serotypes 16 and 18 fused with tpa signal sequence and extracellular domain of Flt3L. Synthetic codon-optimized E6 or E7 genes were fragmented into two parts (C-terminal and N-terminal regions) with a small overlapping sequence (encoding 16 amino acids), and shuffled as shown in Fig. 1a. The fused DNA sequences including tpa, Flt3L and shuffled E6/E7 genes were inserted in high-expression vector, pGX27 (ref. 45), to generate GX-188, which was produced in *E. coli* DH5α under cGMP condition at Althea Technologies, Inc., San Diego, CA.

### Virological and clinical responses

The assessments including colposcopy, histology, endocervical cytology and HPV genotyping test were conducted by local laboratory at the trial site. The assessments were performed in compliance with the standardized method or the internal protocol of Cheil General Hospital and Women’s Healthcare Center. Responses to treatment were evaluated using virology and histology results at weeks 20 and 36 post GX-188E vaccination.

For histological evaluation, biopsy samples were taken during screening and two follow-up visits at weeks 20 and 36. Samples were fixed with 10% formaldehyde and 4–5 μm sections were stained with haematoxylin and eosin. Endocervical samples were collected using cytobrush (Cytyc Corp., Boxborough, MA) during colposcopic examination. This endocervical cytology test was also used in addition to histology for the assessment of GX-188E vaccination. Data from histological and cytological analyses were reviewed independently by at least two pathologists and results were confirmed after discussion with by conference of all pathologists and investigators.

To evaluate virological response, HPV typing was performed to determine whether subjects were infected by either HPV16 and/or HPV18. Samples were collected from the cervix by using a swab-type device, and total DNAs were extracted using the AccuPrep Genomic DNA Extraction kit (Bioneer Com. Seoul, Korea). HPV detection and genotyping was done by Multiplex-PCR system using the IVD CE marked Seeplex HPV4A ACE Screening kit (Seegene Inc., Seoul, Korea) according to the manufacture’s protocol. The Seeplex HPV4A ACE Screening kit can identify HPV16, HPV18, other high-risk types (High risk common: 26, 31, 33, 35, 39, 45, 51, 52, 53, 56, 58, 59, 66, 68, 73 and/or 82), HPV6 and HPV11 types at the same time. PCR products were analysed using an automatic MultiNA instrument (Shimadzu Co., Tokyo, Japan). HPV DNA genotyping was double checked in cervical cells using Cheil HPV DNA Chip with real time PCR to compensate the accuracy of HPV genotype as previously described^56^.

### *Ex vivo* IFN-γ ELISPOT

Cryopreserved and thawed PBMCs were adapted with OpTmizer CTS medium (Life Technologies) for more than 6 h at 37 °C, 5% CO_2_, and subsequently PBMCs (2 × 10^5^ cells per well) were stimulated with 2 μg ml^−1^ of four different pools of HPV16 and HPV18 E6- or E7-derived peptides (20-mer with 10 amino acids overlapping) for 48 h. Phytohaemagglutinin and the medium only served as positive and negative controls, respectively. After stimulation, spots indicating IFN-γ-secreting cells were developed according to manufacturer’s instructions (BD Bioscience). The number of spots was analysed with an automated ImmunoSpot Analyzer (Cellular Technology Ltd.). The HPV-specific responses were calculated by subtracting the mean number of spots in the medium only control from the mean number of spots in experimental wells, which were expressed as SFUs per 10^6^ PBMCs^57^. The assay was performed in triplicate, and the background number of spots was 5.7±2.2 (mean±s.d.). Antigen-specific T-cell responses were considered to be positive when the mean number of spots in the well with the antigen was threefold higher than that of the well with medium control^58^. In addition, a post-analysed vaccine-induced response was defined as positive when at least a threefold increase in T-cell frequency was observed after vaccination compared to before vaccination^15^.

### ICS

Cryopreserved and thawed PBMCs were resuspended in OpTimizer CTS, and rested for more than 6 h at 37 °C, 5% CO_2_. PBMCs were plated in duplicate and stimulated with a combined mixture of HPV16 E6 and E7 peptides in one pool (15-mer with eight amino acids overlapping) at a concentration 2 μg ml^−1^, α-CD3 (positive control, 10 μg ml^−1^, UCHT1, BD Bioscience) or the medium alone (negative control) in the presence of 1 μg ml^−1^ of α-CD28 (L293, BD Bioscience) and α-CD49d (L25, BD Bioscience) for 13 h. Secretion inhibitors (monensin/brefeldin A, BD Bioscience) were added 90 min after initial stimulation. After stimulation, cells were washed with PBS for subsequent immunostaining and polychromatic flow cytometric analysis. Antibodies for staining cells were CD19-APCCy7 (5 μl per test, HIB19, Biolegend), CD4-PerCPCy5.5 (5 μl per test, RPA-T4, Biolegend), CD8-PECy7 (5 μl per test, RPA-T8, BD Bioscience), CD3-BV605 (Bright Violet 605) (5 μl per test, UCHT1, Biolegend), CD3-BV500 (5 μl per test, UCHT1, BD Horizon), Live/dead-APCCy7 (0.5 μl per test, Life technologies), MIP-1β-PE (0.5 μl per test, D21-1351, BD Bioscience), IFN-γ-APC (5 μl per test, 4S.B3, Biolegend), TNF-α-BV421 (5 μl per test, MAb11, Biolegend), IL-2-BV711 (5 μl per test, 5344.111, BD Horizon), CD107a-FITC (5 μl per test, H4A3, BD Bioscience) and CD107b-FITC (5 μl per test, H4B4, BD Bioscience). Fluorescence-activated cell sorting analysis was accomplished by Fortessa flow cytomer (BD Bioscience), and the data were analysed using FlowJo software (Tree Star). Boolean gating was used to determine simultaneous cytokine production from CD8 T cells. Analysis of polyfunctionality was performed with SPICE^59^. A positive response was defined as detecting at least twice the percentage of cytokine-producing T cells than in the medium only control, and the response should be visible as a clearly distinguishable population of cytokine-producing cells separated from the nonproducing cells. A post-analysed vaccine-induced response was defined as detecting at least a threefold increase in the percentage of antigen-specific cytokine-producing T cells than that at prevaccination^60^.

### Cytokine profile analysis by cytometric bead array

Cryopreserved and thawed PBMCs (2 × 10^5^ per well) were resuspended in OpTimizer CTS, and rested for more than 6 h at 37 °C, 5% CO_2_, and subsequently PBMCs were plated in duplicate and were stimulated in RPMI 1,640 containing 10% fetal bovine serum, 100 U ml^−1^ penicillin and 100 μg ml^−1^ streptomycin with a combined mixture of HPV16 E6 and E7 peptides in one pool (15-mer with eight amino acids overlapping) at a concentration of 2 μg ml^−1^ or the medium only as negative control in 96-well plates. Culture supernatants were harvested 48 h after the stimulation and cytokines were quantitated by Th1/Th2/Th17 cytometric bead array (CBA) kit (BD Biosciences). According to manufacturer’s instructions, the proposed detection limit was 2.5–5 pg ml^−1^ (IL-2, IL-4, IL-10, TNF-α and IFN-γ) or 19 pg ml^−1^ (IL-17A), and the cutoff value was set at 5 pg ml^−1^ because the standard curve of each cytokine showed linearity starting at this concentration (Supplementary Fig. 5). Positive antigen-specific reaction was defined as a cytokine concentration above the cutoff value and >2 × the concentration of the medium control^60^. A post-analysed vaccine-induced response was defined as being at least threefold higher in the cytokine production than that at prevaccination^60^.

### Proliferation assay

Cryopreserved and thawed PBMCs (1 × 10^6^ cells per well) were adapted with OpTmizer CTS medium (Life technologies) for more than 6 h at 37 °C, 5% CO_2_. PBMCs were plated and stimulated with a combined mixture of HPV16 E6 and E7 peptides in one pool (15-mer with eight amino acids overlapping) at a concentration 2 μg ml^−1^ in RPMI 1,640 containing 10% fetal bovine serum, 100 U ml^−1^ penicillin and 100 μg ml^−1^ streptomycin for 5 days. α-CD3 mAb and the medium alone served as positive and negative controls, respectively. After 3 days, cell cultures were replaced with 100 μl of fresh medium. At the end of culture, cells were washed with PBS for subsequent immunostaining and polychromatic flow cytometric analysis. The cells were stained with CD19-FITC (5 μl per test, HIB19, Biolegend), CD4-PerCPCy5.5, CD8-PECy7, CD38-BV421 (5 μl per test, HIT2, BD Bioscience), CD3-BV605, Ki-67-PE (20 μl per test, B56, BD Bioscience), and Live/Dead-APCCy7. Responses at least threefold greater than those of the medium control were considered to be positive. A post-analysed vaccine-induced response was defined as being at least threefold higher in the percentage of antigen-specific proliferating CD8 T cells than that at prevaccination.

### Statistical analysis

Descriptive statistics of the safety, pharmacodynamics and pharmacokinetic outcomes was performed using SAS (V9.1) software. Standard and two-tailed paired Student’s *t*-test was performed to analyse statistical significance of all quantitative data using Prism 5.0 software (GraphPad).

## Author contributions

T.J.K., H.-T.J., S.-Y.H., Y.-Y.H., Y.S.S., J.S.P. and Y.C.S. designed study; T.J.K., H.-T.J., H.G.Y., Y.B.S., S.R.H., C.-W.L., S.K., K.S.P., I.-H.L., K.-T.L. and M.S.J. performed experiments and analysed data; T.J.K., H.-T.J., S.-Y.H., Y.B.S., J.-W.W., J.P., C.D.S., Y.S.S., J.S.P. and Y.C.S. wrote the paper.

## Additional information

**How to cite this article**: Kim, T. J. *et al.* Clearance of persistent HPV infection and cervical lesion by therapeutic DNA vaccine in CIN3 patients. *Nat. Commun.* 5:5317 doi: 10.1038/ncomms6317 (2014).

## Supplementary Material

Supplementary Information Supplementary Figures 1-5, Supplementary Tables 1-4, Supplementary Methods and Supplementary References

## Figures and Tables

**Table 1:** Baseline characteristics of the patients.

**Group**	**Patient no.**	**Age at enrolment (years)**	**HLA type**		**Lesion grade**
			**HLA-A**	**HLA-DRB1**	
1 mg cohort	A01	44	*02:06, *30:01	*04:03, *07:01	CIN3, severe dysplasia
	A02	30	*02:06, *02:07	*08:03, *14:07	CIN3, severe dysplasia
	A03	44	*02:01	*01:01, *04:05	CIN3, carcinoma *in situ*
2 mg cohort	A04	37	*26:02, *30:01	*09:01, *16:02	CIN3, carcinoma *in situ*
	A05	23	*02:01, *30:01	*08:03, *13:01	CIN3, severe dysplasia
	A06	25	*02:01, *24:02	*01:01, *09:01	CIN3, severe dysplasia
4 mg cohort	A07	28	*24:02, *26:02	*09:01, *14:06	CIN3, severe dysplasia
	A08	23	*02:01, *24:02	*04:06, *15:01	CIN3, severe dysplasia
	A09	30	*24:02, *26:01	*08:03, *15:02	CIN3, carcinoma *in situ*

CIN3, cervical intraepithelial neoplasia 3; HLA, human leukocyte antigen.

**Table 2:** Virological and clinical responses during and after immunization with GX-188 DNA vaccine by electroporation.

**Patient no.**	**Dose**	**At week 0 (VT1)**			**At week 12 (VT4)**		**At week 20 (VF1)**			**At week 36 (VF2)**		
		HPV status*	**Cytology**	**Histology**	HPV status*	**Cytology**	HPV status*	**Cytology**	**Histology**	HPV status*	**Cytology**	**Histology**
A01	1 mg	16	ASC-H	CIN3	Negative	ASC-US	Negative	NIL	Normal	Negative	NIL	Normal
A02	1 mg	16	HSIL	CIN3	16	HSIL	16	HSIL	CIN3	Negative	NIL	Normal
A03	1 mg	16	HSIL	CIN3	Negative	NIL	Negative	NIL	Normal	Negative	NIL	Normal
A04	2 mg	16	HSIL	CIN3	16	HSIL	16	HSIL	CIN3	ND†	ND†	ND†
A05	2 mg	16 and 18	HSIL	CIN3	16 and common	ASC-US	Negative, common	NIL	Normal	Negative	NIL	Normal
A06	2 mg	16 and common	ASC-H	CIN3	Negative	NIL	Negative	NIL	Normal	Negative	NIL	Normal
A07	4 mg	16 and common	HSIL	CIN3	16 and common	ASC-US	Negative, common	NIL	Normal	Negative‡, common	NIL‡	Normal‡
A08	4 mg	16	ASC-US	CIN3	Negative	NIL	Negative	NIL	Normal	Negative	NIL	Normal
A09	4 mg	16	HSIL	CIN3	16	HSIL	16	HSIL	CIN3	16	HSIL	CIN3

ASC-H, atypical squamous cells-cannot exclude high-grade squamous intraepithelial lesion; ASC-US, atypical squamous cells of undetermined significance; CIN3, cervical intraepithelial neoplasia 3; HPV, human papilloma virus; HSIL, high-grade squamous intraepithelial lesion; NIL, no intraepithelial lesion.

^*^PCR results for the detection of HPV (Negative, both HPV 16 and 18 negative; 16, HPV 16 positive; Common, other high-risk HPV 26, 31, 33, 35, 39, 45, 51, 52, 53, 56, 58, 59, 66, 68, 73 and/or 82 positive).

^†^Not done. A04 patient were treated by cervical conization at week 24.

^‡^A07 patient has visited and undergone examinations for colposcopy and cervical biopsy at week 42 instead of week 36 due to her personal situations.
