# Supplementary material for: Clearance of persistent HPV infection and cervical lesion by therapeutic DNA vaccine in CIN3 patients
Source: Nat Commun. 2014 Oct 30;5:5317. doi: 10.1038/ncomms6317 (PMC4220493; doi:10.1038/ncomms6317)
Supplement: Supplementary Information — Supplementary Figures 1-5, Supplementary Tables 1-4, Supplementary Methods and Supplementary References [file ncomms6317-s1.pdf]

Supplementary Figures

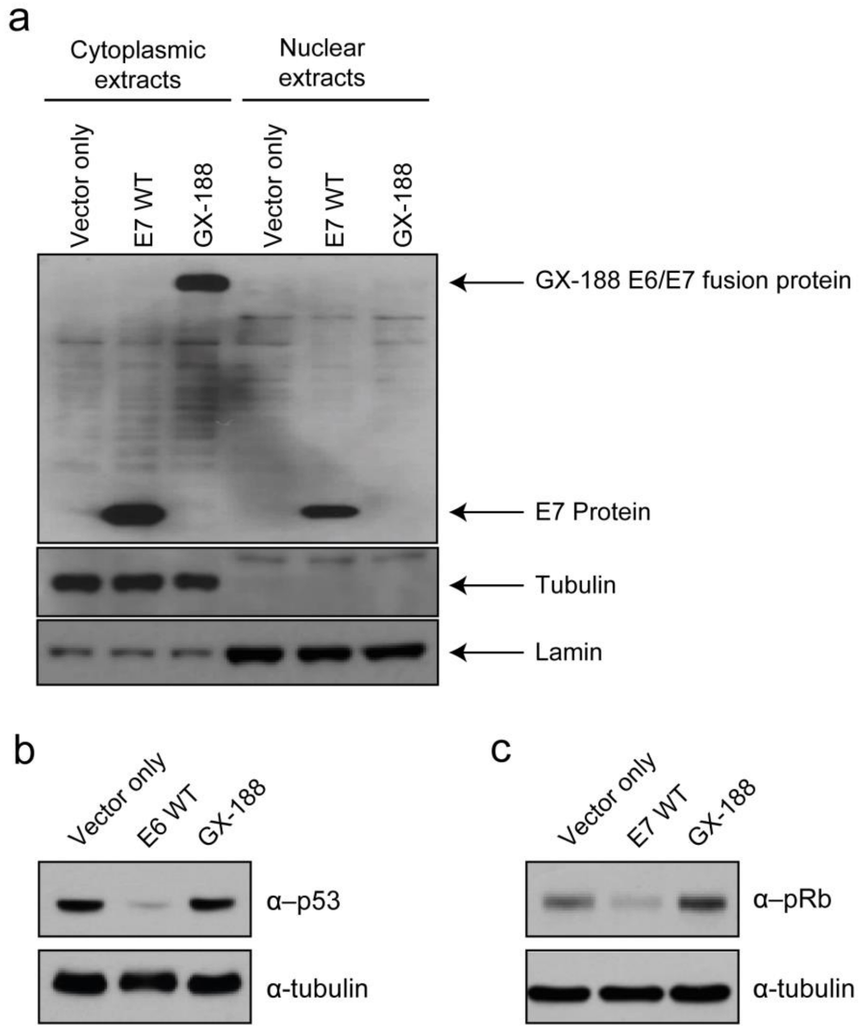

Figure S1 | Subcellular localization of GX-188 E6/E7 fusion protein and its effect on degradation of cellular p53 and pRb proteins. 293T cells were transfected with pGX27 control vector, GX-188, or pGX27 inserted with wild type E6 or E7 genes. Twenty-four hours post transfection, cell lysates were prepared and protein expressions were analyzed by immunoblotting. (a) Cells were resuspended in lysis buffer A (10 mM HEPES, pH 7.9, 10 mM KCl, 0.2 mM EDTA, 1 mM DTT, 0.25 mM PMSF, and proteinase inhibitor cocktail), and the supernatants of extracts were collected as cytoplasmic extracts. The pellet was resuspended in buffer B (20 mM HEPES, pH 7.9, 420 mM NaCl, 2 mM EDTA, 1 mM DTT, 0.25 mM PMSF, and PIC), and their supernatants after pelleting were collected as nuclear

extracts. The purity of the fractions was tested by Western blotting for tubulin and lamin to define the cytoplasmic and nuclear fractions, respectively. (b and c) Cells were resuspended in lysis buffer (20 mM HEPES, pH 7.4, 150 mM NaCl, 5 mM EDTA, 10% glycerol, 0.5% Triton X-100, 1 mM DTT, 1 mM PMSF, 1 mM NaF, 1 mM Na_3Vo_4, and PIC). The supernatants were collected as whole-cell lysates and analyzed for the expression level of cellular p53 and pRb proteins.

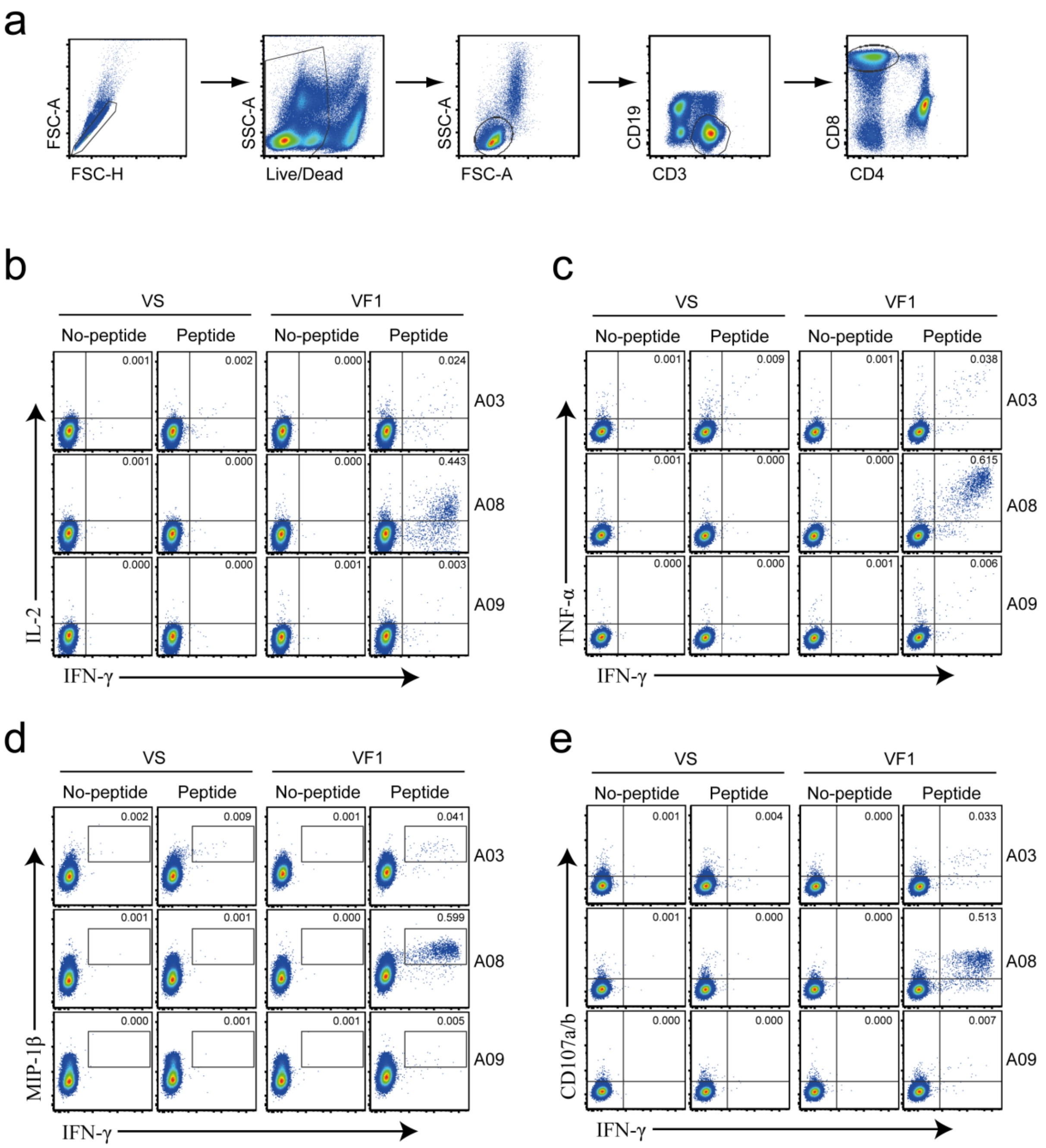

Figure S2 | GX-188E vaccination strongly induced polyfunctional response by HPV16-specific CD8 T cells. Cryopreserved PBMCs of patients harvested before (VS) and after (VF1) GX-188E vaccination were stimulated with a combined mixture of HPV16 E6 and E7 peptide pools for 13 hours and then analyzed with multi-color flow cytometry to simultaneously detect HPV16-specific expression of IL-2, IFN-γ, TNF-α, MIP-1β, and CD107a/b. (a) Gating strategy to identify CD8 T cells by flow cytometry. (b-e) The

representative plots show the frequencies of IFN-γ^+ CD8 T cells co-expressing IL-2 (b), TNF-α (c), MIP-1β (d), and CD107a/b (e) on gated CD8 T cells. The numbers within the plots indicate the frequency of double positive cells among CD8 T cells.

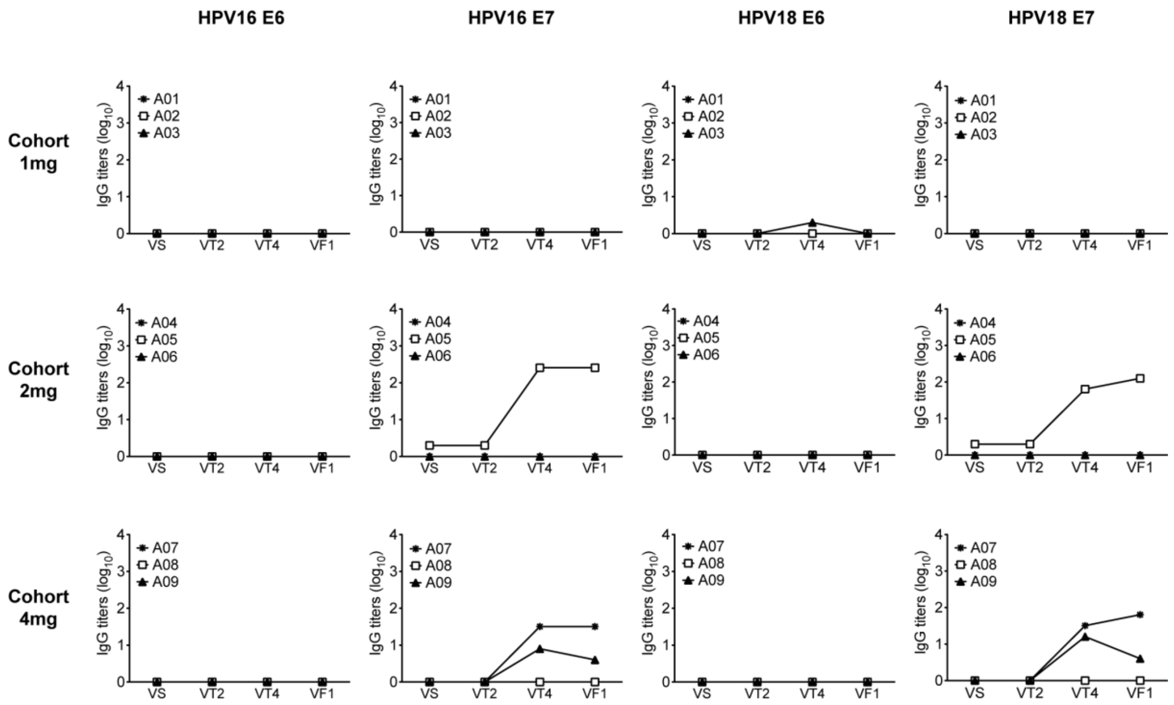

Figure S3 | IgG titer to HPV16/18 E6 and E7 proteins following GX-188E vaccination. Plasma IgG antibody titers against the recombinant E6 and E7 proteins of HPV16 and HPV18 were measured for each patient at a range of dilution by ELISA. Results are shown for each vaccine dose group (1mg, 2mg, and 4mg cohort) prior to immunization (VS), and at the various indicated time points after immunization (VT2, VT4, and VF). Data are represented as dilution fold of samples showing positive signals, which were considered if the average optical density of a sample was greater than the negative cut-off values (0.173 for HPV16 E6, 0.213 for HPV16 E7, 0.214 for HPV18 E6, and 0.227 for HPV18 E7). To determine the background due to non-specific binding, patient's plasma was tested in a well coated with

irrelevant recombinant erythropoietin (EPO) and the resulting optical density of all samples was below negative cut-off value.

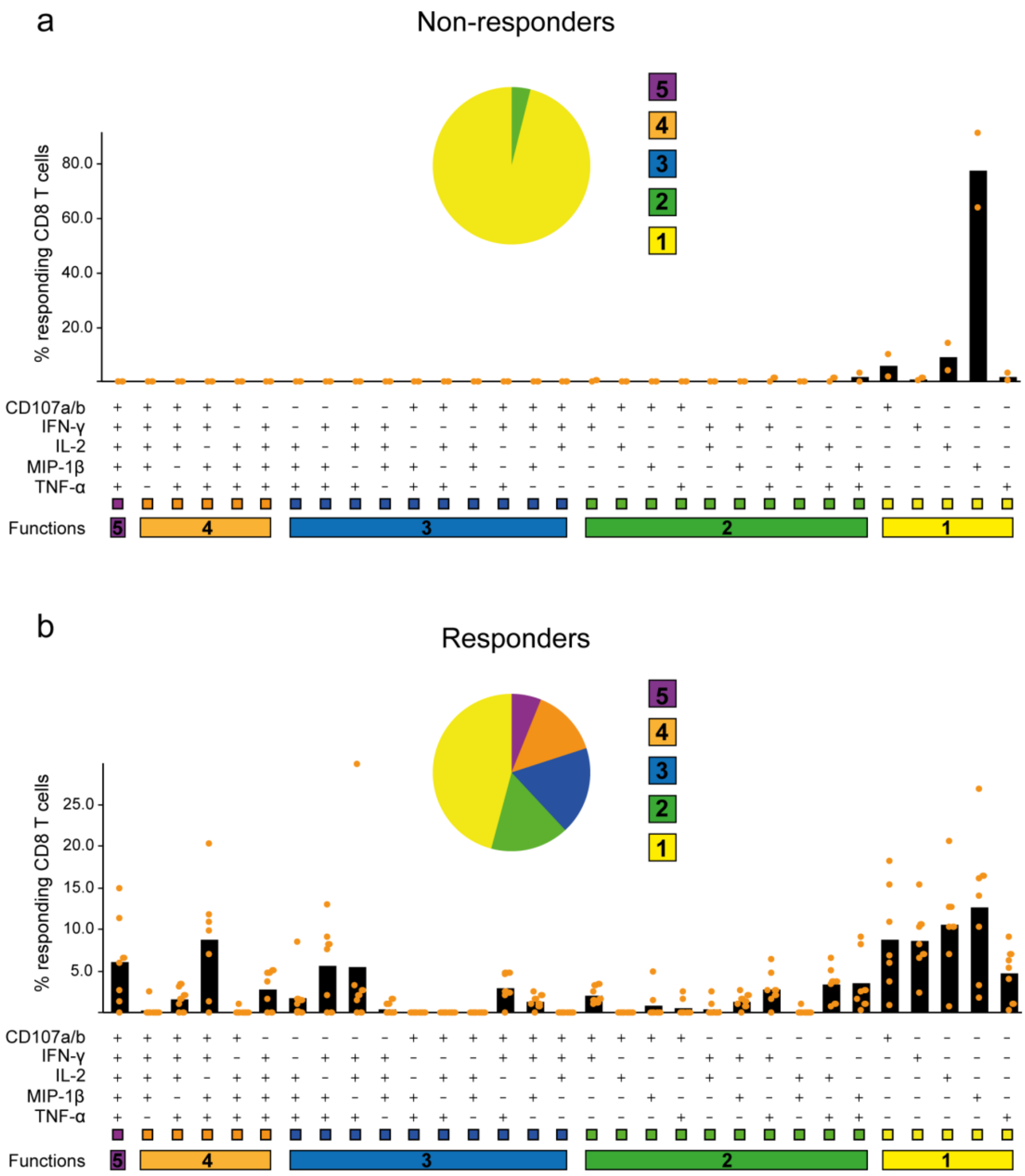

Figure S4 | Polyfunctional profiles of HPV-specific CD8 T cells induced by GX-188E vaccine in the non-responders and responders groups of GX-188E vaccination. The frequency of HPV16-specific IL-2-, IFN-γ-, TNF-α-, MIP-1β-, or CD107a/b-producing CD8 T cells was measured 20 weeks (VF1) after vaccination using Boolean gating. The individual

data from the patients were pooled together and grouped into non-responders (A04 and A09) and responders (A01, A02, A03, A05, A06, A07 and A08) according to clinical and virological outcomes. The non-responders' (a) and responders' (b) polyfunctional CD8 T-cell responses to HPV16 E6/E7 peptides post GX-188E vaccination are presented as the graph and pie chart. In the graph, black bars represent mean response, and orange dots correspond to the response from a single subject. All the possible functional combinations of effector functions are listed along the *x*-axis. The five horizontal bars of different colors below *x*-axis depict the populations of five (purple), four (orange), three (blue), two (green) or one (yellow) functional responses. The pie chart represents the relative frequency of HPV16 E6/E7-specific CD8 T cells with each combination of the 5 functional responses.

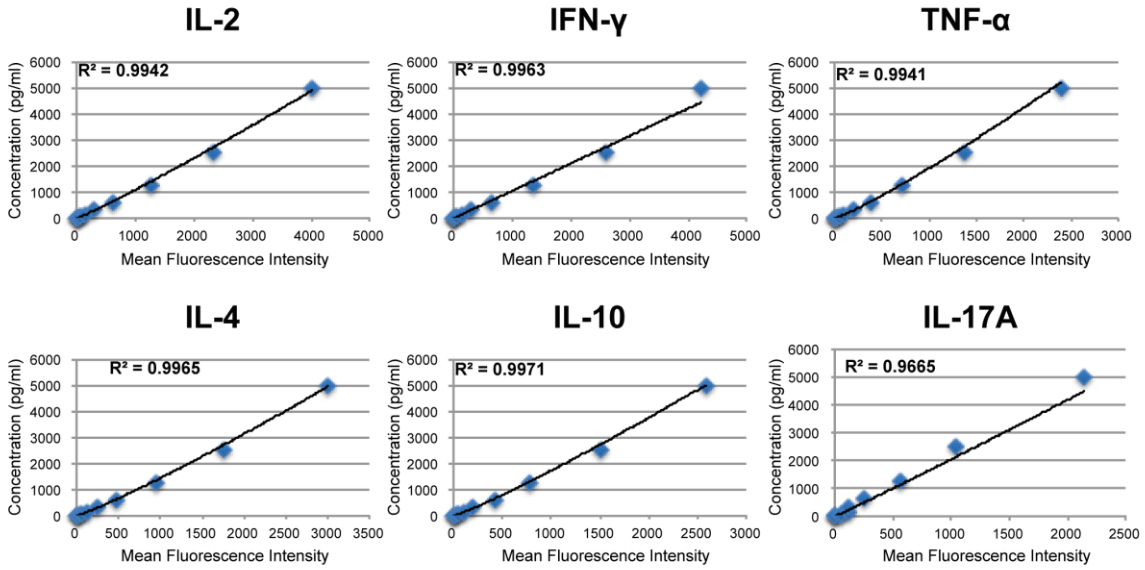

Figure S5 | Th1/Th2/Th17 subset cytokine standards generated by cytometric bead array. To ensure a valid analysis of protein below 10 pg ml^{-1} (the default outlined limit for quantification), human Th1/Th2/Th17 cytokine standards were reconstituted in 50 µl assay diluent, and the standards were constructed from 5 - 5,000 pg ml^{-1} (dilution rate; 1:1, 1:2, 1:4,

1:8, 1:16, 1:32, 1:64, 1:128, 1:256, 1:512, and 1:1028). Cytokine standard curves were generated after sample acquiring using power fit and R^2>0.96 for all cytokines. The concentration for each cytokine in cell supernatants was determined by interpolation from the corresponding standard curve.

Supplementary Tables

Supplementary Table 1. Adverse drug reactions classified by MedDRA System Organ Class (SOC) during the clinical study

Adverse drug reactions	1 mg (n=3)	2 mg (n=3)	4 mg (n=3)
General disorders and administration site conditions			
Chills	0	1 [1]	0
Fatigue	0	0	2 [2]
Injection site erythema	0	1 [1]	1 [1]
Injection site pain	1 [2]	2 [5]	2 [6]
Injection site paraesthesia	1 [1]	0	0
Swelling	0	1 [1]	0
Infections and infestations			
Rhinitis	0	0	1 [1]
Nervous system disorders			
Headache	0	0	2 [4]
Hypoaesthesia	0	1 [1]	0

[a] Data are presented as a number of subjects and a number of incidences ([ ])

All adverse events possibly related to DNA vaccine plus electroporation, or unknown (fatigue, rhinitis, headache) were indicated. CTCAE grades of the events are 1 (mild), and all events recovered completely within 3 days after injection.

Supplementary Table 2. Summary of Hematology test

		1 mg (n=3)			2 mg (n=3)			4 mg (n=3)			Total (n=9)		
WBC	VS	5.2	±	1.3	5.1	±	0.6	4.5	±	0.7	4.9	±	0.9
(10^9 L^{-1})	VT2	5.7	±	1.7	4.2	±	0.1	4.4	±	0.5	4.8	±	1.1
	VT4	5.6	±	0.8	3.9	±	0.3	4.3	±	0.2	4.6	±	1.0
	VF1	6.0	±	1.2	4.1	±	0.7	5.8	±	0.3	5.3	±	1.1
Neutrophils	VS	51.3	±	1.4	59.6	±	13.2	55.7	±	14.6	55.5	±	10.5
(%)	VT2	55.6	±	5.1	55.7	±	8.2	48.6	±	9.1	53.3	±	7.5
	VT4	51.2	±	5.0	54.4	±	3.5	49.5	±	9.6	51.7	±	6.1
	VF1	53.5	±	8.1	52.5	±	10.3	49.3	±	12.6	51.7	±	9.3

Lymphocytes	VS	38.2	±	2.5	29.5	±	11.2	35.8	±	14.5	34.5	±	10.0
(%)	VT2	35.2	±	4.4	30.9	±	8.7	42.2	±	8.9	36.1	±	8.3
	VT4	39.7	±	3.7	31.2	±	4.8	41.1	±	10.5	37.3	±	7.6
	VF1	36.4	±	5.6	33.3	±	7.9	42.1	±	11.7	37.3	±	8.5
Monocytes	VS	5.0	±	0.9	6.1	±	1.1	4.7	±	0.2	5.2	±	1.0
(%)	VT2	4.6	±	0.5	7.3	±	1.2	4.2	±	1.2	5.4	±	1.7
	VT4	4.6	±	0.3	8.0	±	2.4	3.8	±	0.2	5.5	±	2.3
	VF1	5.0	±	1.1	8.3	±	1.4	4.0	±	0.4	5.8	±	2.1
Eosinophils	VS	2.5	±	0.7	2.1	±	0.7	1.4	±	0.4	2.0	±	0.7
(%)	VT2	2.1	±	0.2	2.5	±	1.0	2.0	±	1.0	2.2	±	0.7
	VT4	2.1	±	1.0	3.1	±	0.8	2.6	±	1.9	2.6	±	1.2
	VF1	2.2	±	1.3	2.8	±	0.4	1.6	±	0.7	2.2	±	0.9
Basophils	VS	0.6	±	0.2	0.4	±	0.1	0.4	±	0.2	0.5	±	0.2
(%)	VT2	0.4	±	0.1	0.4	±	0.2	0.5	±	0.1	0.4	±	0.1
	VT4	0.5	±	0.3	0.6	±	0.3	0.4	±	0.1	0.5	±	0.2
	VF1	0.4	±	0.1	0.3	±	0.2	0.6	±	0.2	0.4	±	0.2
RBC	VS	4.0	±	0.1	4.2	±	0.2	4.2	±	0.2	4.1	±	0.2
(10^{12} L^{-1})	VT2	4.0	±	0.1	4.2	±	0.1	4.3	±	0.2	4.2	±	0.2
	VT4	4.0	±	0.1	4.5	±	0.0	4.3	±	0.3	4.3	±	0.3
	VF1	4.0	±	0.1	4.3	±	0.2	4.2	±	0.0	4.2	±	0.2
Hemoglobin	VS	12.6	±	0.4	12.7	±	1.6	13.1	±	0.4	12.8	±	0.9
(g dL^{-1})	VT2	12.5	±	0.2	12.4	±	1.6	13.0	±	0.7	12.7	±	0.9
	VT4	12.4	±	0.8	12.6	±	2.0	12.9	±	0.6	12.6	±	1.1
	VF1	12.3	±	1.1	12.2	±	1.8	13.0	±	0.1	12.5	±	1.1

Data are presented as a mean value ± s.d.

Supplement Table 3. Change of Flt3L concentration in blood

Time points	1 mg (n=3)	2 mg (n=3)	4 mg (n=3)
VS	88.23 ± 26.93	63.70 ± 10.87	75.59 ± 19.29
VT2	103.00 ± 43.08	62.32 ± 6.86	76.46 ± 5.97
VT4	88.71 ± 17.80	66.52 ± 5.01	77.41 ± 19.90
VF1	93.86 ± 38.59	72.38 ± 10.96	91.20 ± 4.80
VF2	80.04 ± 29.15	71.71 ± 8.10	80.89 ± 16.00

Data are presented as a mean value ± s.d. (pg ml^{-1})

Supplementary Table 4. Undetectable levels of anti-ds DNA antibody in subjects

	A01	A02	A03	A04	A05	A06	A07	A08	A09
VS (IU ml^{-1})	<10.0	<10.0	<10.0	<10.0	<10.0	<10.0	<10.0	<10.0	<10.0
VF1 (IU ml^{-1})	<10.0	<10.0	<10.0	<10.0	<10.0	<10.0	<10.0	<10.0	<10.0

Detection limit, 10 IU ml^{-1}

Supplementary Methods

Cell fractionation and immunoblotting.

The 293T cells were transfected with an expression plasmid encoding GX-188 or wild type E6 or wild type E7. For whole-cell protein lysates, cells were resuspended in lysis buffer (20 mM HEPES, pH 7.4, 150 mM NaCl, 5 mM EDTA, 10% glycerol, 0.5% Triton X-100, 1 mM DTT, 1 mM PMSF, 1 mM NaF, 1 mM Na_3Vo_4, and PIC). Nuclear and cytoplasmic fractions of cells were prepared as follows : cells were washed once with ice-cold phosphate-buffered saline (PBS) and collected at 3,000 rpm for 5 min. Cells were resuspended in buffer A (10 mM HEPES, pH 7.9, 10 mM KCl, 0.2 mM EDTA, 1 mM DTT, 0.25 mM PMSF, and proteinase inhibitor cocktail). After incubation on ice for 5 min, NP-40 was added to a final concentration of 0.25%. The mixtures were vortexed at high speed for 10 seconds. Extracts were collected by centrifugation at 13,000 rpm for 30 seconds. The supernatants were collected as cytoplasmic extracts. The pellet was resuspended in buffer B (20 mM HEPES, pH 7.9, 420 mM NaCl, 2 mM EDTA, 1 mM DTT, 0.25 mM PMSF, and PIC), followed by incubation at 4°C for 30 min under gentle agitation. The mixtures were spun at 13,000 rpm for 15 min, and the supernatants were collected as nuclear extracts. The following antibodies were used: anti-HPV 16 E6 (N-17), anti-HPV 16 E7 (ED17), anti-p53 (FL-393), anti-pRb (C-15) antibodies were purchased from Santa Cruz Biotechnology, Inc. Anti-Lamin B1, anti-β-tubulin antibodies were purchased from Abcam.

Flt3L ELISA.

The level of Flt3L in the blood was measured using Flt3L ELISA kit (DFK00, R&D Systems) according to the manufacturer's instructions. Briefly, plasma samples and standards were

added in microplate coated with a monoclonal antibody specific for human Flt3L. After washing away any unbound substances, an enzyme-linked polyclonal antibody specific for human Flt-3 Ligand was added to the wells. Following a wash to remove any unbound antibody-enzyme reagent, a substrate solution was added to the wells. The color development was stopped by adding 2N sulfuric acid and the intensity of the color was measured using microplate reader (Molecular devices, SpectraMax plus 384). Level of Flt3L in the blood (pg per ml) was calculated by creating standard curve using computer software capable of generating a log/log curve-fit (SoftMax Pro Software, v5.4.1). Data are presented as a mean value ± s.d. of triplicate samples.

Detection of anti-ds DNA antibody in subjects.

The level of anti-ds DNA antibody was determined by ELISA (CHORUS dsDNA-G, DIESSE, Italy) in LabGenomics Co. Ltd., Korea. Briefly, the plasma (50 μl) was added into the microplate well coated with purified human DNA, and then, after washing, incubation was performed with anti-human IgG antibody conjugated with horse radish peroxidase. The unbound conjugate was eliminated, and the TMB substrate was added. To check the validity of the results, control samples supplied with the kit were used. If the signal for the control sample has a value outside the acceptable range, the calibration should be repeated. The calibration range was 10.0-150.0 IU ml^{-1}. The test sample can be interpreted as follows; positive when the result is > 30.0 IU ml^{-1}, negative when the results is < 20.0 IU ml^{-1}, doubtful for all values between 20.0 and 30.0 IU ml^{-1}. In the case of a doubtful result, the test should be repeated. Diagnostic sensitivity, cross-reactions, specificity, and precision of the test were described in the kit manual. The limit of detection was 10 IU ml^{-1}.

ELISA for the titration of HPV16/18 E6- and E7-specific IgG antibodies.

Plasma samples were collected and frozen at -70 °C. A binding ELISA was performed to measure the anti-HPV16/18 E6 or E7 antibody response induced by GX-188E vaccination. Endpoint titers of antibodies were determined by coating 96-well enzyme immunoassay plates (Thermo Scientific™) with HPV16/HPV18 E6 or E7 proteins (1μg ml^{-1}) (recombinant HPV16 E6, HPV16 E7, and HPV18 E7 were purchased from ProteinX Lab; recombinant HPV18 E6 was purchased from MyBioSource). The plate was blocked with PBS, 5% skim milk for 1 hour at room temperature. Test plasma were serially diluted in PBS containing 5% skim milk and 0.1% Tween 20, and added to the plate wells in triplicate. After 1 hour incubation at room temperature, E6- or E7-specific antibodies were detected by incubating the plates for 1 hour at room temperature with goat anti-human IgG antibody conjugated to HRP (Bethyl, A80-104P). After a last wash (Tablet, Fluka), specific binding was detected with TMB substrate (SurModics). The reaction was stopped with 0.5N H_2SO_4 (Sigma-Aldrich), and the absorbance read at 450nm in a microplate reader (Molecular devices, SpectraMax plus 384). Negative cut-off (NCO) values were defined as the mean optical density plus 1.645 × s.d. of 12 healthy control plasma (Biochemed)[1]. Positivity was considered if the average optical density of a sample was greater than NCO values (0.173 for HPV16 E6, 0.213 for HPV16 E7, 0.214 for HPV18 E6, and 0.227 for HPV18 E7). To account for non-specific binding of samples to the plate, each plasma was tested in a well coated with an irrelevant protein, EPO-BRP (EDQM, batch 3, ph. Eur. Reference standard).

Supplementary References

1. Mire-Sluis AR, *et al.* Recommendations for the design and optimization of

immunoassays used in the detection of host antibodies against biotechnology products. *Journal of immunological methods* **289**, 1-16 (2004).
